# Diversity and abundance of ammonia oxidizing archaea in tropical compost systems

**DOI:** 10.3389/fmicb.2012.00244

**Published:** 2012-07-09

**Authors:** Vidya de Gannes, Gaius Eudoxie, David H. Dyer, William J. Hickey

**Affiliations:** ^1^Faculty of Science and Agriculture, Department of Food Production, The University of the West IndiesSt. Augustine Campus, Republic of Trinidad and Tobago; ^2^O. N. Allen Laboratory for Soil Microbiology, Department of Soil Science, University of Wisconsin-MadisonMadison, WI, USA

**Keywords:** ammonia oxidizing archaea, compost, diversity, molecular ecology, nitrification

## Abstract

Composting is widely used to transform waste materials into valuable agricultural products. In the tropics, large quantities of agricultural wastes could be potentially useful in agriculture after composting. However, while microbiological processes of composts in general are well established, relatively little is known about microbial communities that may be unique to these in tropical systems, particularly nitrifiers. The recent discovery of ammonia oxidizing archaea (AOA) has changed the paradigm of nitrification being initiated solely by ammonia oxidizing bacteria. In the present study, AOA abundance and diversity was examined in composts produced from combinations of plant waste materials common in tropical agriculture (rice straw, sugar cane bagasse, and coffee hulls), which were mixed with either cow- or sheep-manure. The objective was to determine how AOA abundance and diversity varied as a function of compost system and time, the latter being a contrast between the start of the compost process (mesophilic phase) and the finished product (mature phase). The results showed that AOA were relatively abundant in composts of tropical agricultural wastes, and significantly more so than were the ammonia-oxidizing bacteria. Furthermore, while the AOA communities in the composts were predominatly group I.1b, the communities were diverse and exhibited structures that diverged between compost types and phases. These patterns could be taken as indicators of the ecophysiological diversity in the soil AOA (group I.1b), in that significantly different AOA communties developed when exposed to varying physico-chemical environments. Nitrification patterns and levels differed in the composts which, for the mature material, could have significant effects on its performance as a plant growth medium. Thus, it will also be important to determine the association of AOA (and diversity in their communities) with nitrification in these systems.

## Introduction

Composting is a self-heating process wherein microbes are employed to transform organic wastes into humus-like materials. The composition and dynamics of microbial communities in compost have been examined extensively, but largely with a focus on heterotrophic microbes and their activities related to carbon transformations (Tuomela et al., 2000; Takaku et al., 2006). These studies have shown that microbial community successions accompany the physico-chemical evolution of composts through their characteristic three stages (mesophilic-, thermophilic- and mature-phases), and that the composition of the microbial communities are affected by the types of materials used in the compost as well as the compost conditions (Peters et al., 2000; Ryckeboer et al., 2003; Charest et al., 2004; Anastasi et al., 2005; Tiquia, 2005; Klamer and Baath, 2006; Partanen et al., 2010)

Transformations of nitrogen (N) are important in composting for supporting biodegradative processes, and include ammonification and nitrification, both of which are microbially-mediated. Organic N in the fresh substrates can be released as ammonium *via* ammonification (Bernal et al., 2009), and ammonium can subsequently be oxidized to nitrate *via* nitrification (Bernal et al., 2009; Maeda et al., 2011). Nitrification rates and nitrate levels play significant roles in affecting the fate and behavior of N in composts. These roles include assimilation into microbial biomass/humus, leaching by saturated flow and gaseous N oxide emissions which are produced *via* nitrification and denitrification (Rudolf and Kroneck, 2005; Maeda et al., 2011). Nitrification is thus particularly important in the finished compost product (mature phase) as it greatly affects the availability of inorganic N forms to plants (Smiths et al., 2010). Yet, there is little information concerning the diversity and community dynamics of nitrifying microbes in composts.

A key step in nitrification is oxidation of ammonia to nitrite, which had been regarded as the sole domain of the ammonia oxidizing bacteria (AOB). But, the recent discovery of ammonia oxidizing archaea (AOA) has challenged that paradigm, and called for re-examination of nitrifying communities (Di et al., 2010; Gubry-Rangin et al., 2010, 2011; Zhang et al., 2010; Herrmann et al., 2011; Li et al., 2011). Reports concerning AOA in composts have been limited to two studies. In one of these studies, Yamamoto et al. (2010) examined communities of AOB and AOA in a cattle manure compost. In this system, AOB initially out-numbered AOA but, by the mature phase, AOA were predominant. Separation by denaturing gradient gel electrophoresis (DGGE) of archaeal *amoA* PCR products gave two bands with no significant change over time (Yamamoto et al., 2010). A total of 31 archaeal *amoA* sequences were obtained from these bands, which were mostly identical or nearly so. Thus, while AOA communities appeared to grow during the course of composting, the AOA community had relatively low diversity with no detectable alteration. In the other study, (Zeng et al., 2011) followed a similar approach to analyze AOB and AOA communities in several agricultural wastes, and their DGGE analyses showed AOA communities to be relatively diverse and dynamic in structure. But, while 21 bands were resolved by DGGE of archaeal *amoA* PCR products, no sequence data was reported. Thus, the phylogenetic diversity was uncertain.

In the present study, AOA abundance and diversity was examined in composts produced from combinations of crop waste materials common in tropical agriculture (rice straw, sugar cane bagasse and coffee hulls), which were mixed with either cow- or sheep-manure. The objective was to determine how AOA abundance and diversity varied as a function of compost system and time, the latter being a contrast between the start of the composting process (mesophilic phase) and the finished product (mature phase).

## Materials and methods

The study followed a randomized, 3 × 2 factorial design with six treatments and two replications per treatment. Agricultural plant waste materials used for composting were rice straw, sugar cane bagasse and coffee hulls (Table 1), which were amended with either cow- or sheep-manure (Table 1) to obtain the desired C:N of 25–35 (Golueke, 1992). All materials to be composted were air dried for 5 d prior to shredding and mixing. The desired C: N was achieved based on a volume ratio. Rice straw and manures were mixed in the ratio of 5:1, sugar cane bagasse and manures were mixed in the ratio of 3:1 and coffee hulls and manures were mixed in the ratio 10:1. Each compost type consisted of 0.218 m^3^ of material that was placed into in-vessel composting units. The latter were rotatable, plastic drums (length, 92 cm; diameter, 61 cm; volume, 0.268 m^3^) with built-in mixing paddles, mounted upon 50 cm-tall brackets. All vessels were housed for the duration of the study in a greenhouse at the Department of Food Production, University West Indies, St. Augustine Campus, Trinidad and Tobago. The composts were aerated every 3–4 d by vessel rotation. Composts were sampled at day 0, at 5 d intervals during the study, and at the conclusion of the experiment on day 82 (week 12). At each sampling time, a flame-sterilized stainless steel forceps was used to take *ca*. 33 g of material from a depth of *ca*. 30 cm at six points within the pile. All six of these samples were pooled in a sterile Nasco Whirl Pak bag (Lakewood Biochemical Co., Inc. Dallas, Texas), mixed and then a 50 g subsample transferred to a separate Whirl Pak bag, which was stored at −20°C for DNA extraction. The remainder of the material was used for physiochemical analyses described below.

**Table 1:** **Selected physico-chemical characteristics of substrates used in compost construction^a^**.

**Substrate**	**TOC (mg/kg)**	**TN (mg/kg)**	**BD (Mg/m^3^)**	**pH**	**EC (S/m)**	**WHC (%)**	**M (%)**	**LI (%)**	**CE (%)**	**AMM (mg/kg)**	**NIT (mg/kg)**	**Fe**	**Cu**	**Zn**	**K**	**P**	**Mn**
												**μg/g**					
Rice Straw	506	10.4	0.09	6.9	2.2× 10^−3^	101	51	6.1	43	28.5	19.2	13.6	0.2	2.7	819	125	36.6
Sugarcane bagasse	549	2.8	0.04	4.6	0.438× 10^−3^	157	52	30.1	42	27.8	17.5	17.8	0.1	250	960	85	9.9
Coffee hulls	508	15.1	0.24	4.5	6.5× 10^−3^	151	17	27.7	30	30.6	10.0	21.2	9.0	759	337	1466	10.4
Cow Manure	348	20.2	0.45	7.1	1.88× 10^−3^	65	57	ND	ND	50.3	32.7	13.9	0.3	970	780	475	8.1
Sheep Manure	361	16.3	0.50	7.3	10.0× 10^−3^	78	58	ND	ND	51.2	33.2	14.2	0.4	880	800	480	7.8

a Abbreviations: TOC = Total Organic Carbon, TN = Total Nitrogen, BD = Bulk Density, EC = Electrical Conductivity, WHC = Water Holding Capacity, M = Moisture, Li = Lignin, CE = Cellulose, AMM = Ammonium, NIT = Nitrate, ND = Not Determined.

### Physico-chemical analyses of compost material

Compost temperature was measured with a long stem temperature probe (ThermoWorks, Lindon, UT) that was inserted at the center of the pile to a depth of *ca*. 30 cm (Takaku et al., 2006). The probe was withdrawn, and then inserted sequentially into two additional spots at the piles center to acquire a total of three readings. Temperature was measured daily between 10:00 AM and 10:30 AM. Moisture content was also monitored daily by the “squeeze test” method (Thompson et al., 2003). The composite compost samples were analyzed for moisture, pH, temperature, electrical conductivity (EC), total nitrogen (TN), ammonium, nitrate and total organic carbon (TOC). For moisture content, samples (25 g wet weight, *M*_*i*_) in pre-weighed containers were placed in a Model 40 GC lab oven (Quincy Inc, Chicago, IL) held at 70°C. Upon reaching a constant weight (M_f_) the percent water content was calculated as: [(*M*_*i*_–*M*_*f*_)/*M*_*f*_] × 100 (Thompson et al., 2003). The samples were next dried, ground with a Wiley Mill (Thomas Model 4, Swedesboro, NJ) then analyzed for TOC and TN. For TOC, the Loss on Ignition technique was used (Thompson et al., 2003) and samples (triplicates, 10 g dry weight) in pre-weighed crucibles were placed in a muffle furnace (Model 184 A, Swedesboro, NJ) that was slowly heated to a holding temperature of 550°C. After 2 h incubation, samples were cooled in a desiccator, and then TOC was calculated by dividing the organic matter content by “Van Bemmelen factor” of 1.724 (Nelson and Sommers, 1996). The Kjeldahl method was used to determine TN, as well as ammonium and nitrate (Thompson et al., 2003). For EC and pH determinations, sample-water slurries (1:5, w/v; sample: distilled deionized water) were mixed with a wrist action shaker (Model 75, Burrell Scientific Pittsburgh, PA) for 20 min at 180 RPM, and then filtered through Whatman one membranes. Measurements of EC and pH were taken for each clarified sample with an Eijkelkamp pH/mV/EC/Salinity/T/02 m (Agrisearch Equipment ZG Giesbeek, the Netherlands). The apparent nitrification rate was calculated as the difference in nitrate concentration between two consecutive weekly measures (e.g., [nitrate]_wk2_ − [nitrate]_wk1_).

Statistical analyses of physico-chemical parameters were carried out using Repeated ANOVA measurements and significance was determined at the 95% confidence interval. GenStat 13 software was used (VSN International, Hemel Hempstead, HP1 1ESUK).

### DNA extraction, clone library construction, DNA sequencing and quantitative PCR (qPCR)

A Power Soil DNA Isolation kit (MO BIO Laboratories, Inc. West Carlsbad, CA) was used to extract DNA from compost samples, and post-extraction clean-up was done with the Power Clean DNA Clean up kit (MO BIO Laboratories), with both procedures following the manufacturer's instructions. Each of the 50 μl extracts were then dried by using a DNA speedvac (Thermo Scientific) and resuspended in 50 μl of sterile distilled deionized water. DNA extracts were prepared from the day 0 (mesophilic stage), day 2 (thermophilic stage for rice), day 3 (thermophilic stage for coffee) and day 82 (designated mature stage) samples of each replicate of each treatment. Aliquots of the mesophilic and day 82 treatment replicates were pooled, and a sample of the pooled extract was used for PCR. Amplification of archaeal *amo*A was then done by using the FailSafe Enzyme System (Buffer E, Epicentre Technology, Madison and WI) with primers Arch-amoA–1F (5′- STAATGGTCTGGCTTAGACG-3′) and Arch-amoA–2R (5′-GCGGCCATCCATCTGTATGT-3′) and the thermal cycling protocol described by primers designer (Francis et al., 2005). An Eppendorf MasterCycler (Eppendorf, Hauppauge, NY) was used for PCR and all reactions were done in 25 μl total volume containing 2 μl of DNA extract. The PCR products were then purified using the QIAquick® PCR Purification Kit (QIAGEN, Germantown, MD, USA) according to the manufacturer's instructions.

Purified PCR products were ligated into pGEM T-Easy (Promega, Madison, WI) according to the manufacturer's protocol, and ligation products transformed into *E.coli* JM 109 competent cells (Promega, USA). Transformants were then selected by plating on Luria Bertani medium containing ampicilin and X-gal (each at 100 μg ml^−1^), and libraries subsequently created by randomly picking white colonies. A total of ten libraries were made from the following treatments at both the mesophilic and thermophilic stages: rice straw + sheep manure, bagasse + cow manure, bagasse + sheep manure, coffee hulls + cow manure, coffee hulls and sheep manure The treatment with rice straw and cow manure was omitted for logistical reasons. Clones were then sequenced by using primer Arch-amoA-1F and the BigDye Terminator Cycle Sequencing system (Applied Biosystems, Foster City, CA). Reactions were analyzed with an Applied Biosystems 3730×l DNA analyzer available at the University of Wisconsin-Madison, Biotechnology Center.

For analysis of AOA and AOB population densities by qPCR, DNA extracts used for clone library construction were analyzed (day 0, mesophilic stage; day 82, mature stage) along with extracts made from the thermophilic phases of the rice (day 2) and coffee (day 3) systems. Primers for qPCR were designed to give amplicons of 50–150 bp for optimal amplification efficiency (Arezi et al., 2003). The primers used for *amoA* clone library construction yielded an amplicon of 635 bp. For archaeal *amoA*, oligonucleotides were selected from alignment of all currently available *bone fide* archaeal *amoA* sequences (EU239961, FR773159, JN227489, HM345609, HM345611, HM345608, HM345610). Primers for bacterial *amoA* were designed from alignment of selected cultured isolates (U76552, U76553, AF327919, NC_008344, NC_004757, NC_007614, NC_015731). For quantification of archaeal *amoA*, the primer pair used was (primer name, T_m_) 5′-YACTGACTGGRSKTGGACATCSTT-3′ (amoA_AF, 60.24°C) and 5′-GTBGCTGTW CCHGGAACACCTGT-3′ (amoA_AR, 61.44°C). PCR Primers used for quantification of bacterial *amoA* were: 5′-CTGGCRGGWGACTGGGAYTTCTGG-3′ (amoA_BF, 63.35°C) and 5′-SAGGCGRTAGTTBACCCACAAGTA-5′ (amoA_BR, 58.79°C). These primer pairs gave archaeal *amoA* and bacterial *amoA* amplicons of 111- and 126-bp, respectively. In each experiment, two independent master mixes were prepared for the standard curve as well as the DNA to be analyzed, which included the primers (2 μM final concentration each) and 1X iQ SYBR green Supermix (BioRad Laboratories, Inc., Hercules, CA). The templates used in standard curve generation were the archaeal *amoA* and bacterial *amoA* amplicons inserted into pGem T-Easy (Promega, Madison, WI). The standard curves were prepared with serial dilutions of the template spanning the range of 10^−3^ to 10^−9^, and had *r*^2^ values of 98–99.6%. Amplification efficiency was 93–96%. PCR was done with a MyiQ Real-Time qPCR detection system (Bio-Rad). The PCR reaction was prepared in a volume of 50 μl, and run as a set of triplicates (each 15 μl total volume). Each DNA sample was amplified by using the following thermal cycling protocol: 95.0°C for 3 min; 40 cycles of denaturation at 95.0°C for 10 s, annealing at 59.0°C for 45 s. A melting-curve protocol began after amplification and consisted of 1 min at 95.0°C, followed by 1 min at 55 and 80°C 10 s steps with a 0.5°C increase in temperature at each step. The R programming environment (http://www.R-project.org) was used to analyze qPCR data sets by ANOVA and pair-wise comparisons with the Tukey-Kramer method.

### Bioinformatics and phylogenetic analyses

Raw sequence files were imported into Geneious Pro™ 5.4 (Biomatters Ltd., Auckland 1010, New Zealand) manually curated, aligned and then trimmed to uniform length of 500 bp. The library was examined for potential chimeric sequences with UCHIME [(Erguder et al., 2009), http://www.drive5.com/uchime/] using the database of all currently available *bone fide* archaeal *amoA* sequences (see above). Sequence identities were determined by searching GenBank with the BLAST-N web server (http://www.ncbi.nlm.nih.gov/). Phylogenetic tree creation was done with Geneious Pro™ v. 5.4.5, pairwise sequence alignments were assembled with ClustalW (http://www.clustal.org/) and trees constructed by the neighbour-joining method and the Jukes-Cantor distance model. The bootstrap re-sampling method was used with 1000 replicates. Phylogenetic trees were illustrated with FigureTree v. 1.3.1 (http://tree.bio.ed.ac.uk/software/figtree). The determinations of operational taxonomic units (OTU) and rarefaction analyses were done with Mothur (http://www.mothur.org, v. 1.22.1; Schloss et al., 2009). Sequence alignment and distance files used in Mothur analyses were created with ClustalW and PHYLIP v. 3.69 (http://evolution.genetics.washington.edu/phylip.htm), respectively. Principle component analyses (PCA) were done with Fast Unifrac *via* webserver (http://bmf.colorado.edu/fastunifrac/).

### Sequence accession numbers

Equences obtained in this study are deposited in Genbank under accession numbers JX026069-026277.

## Results

**Figure 1:**
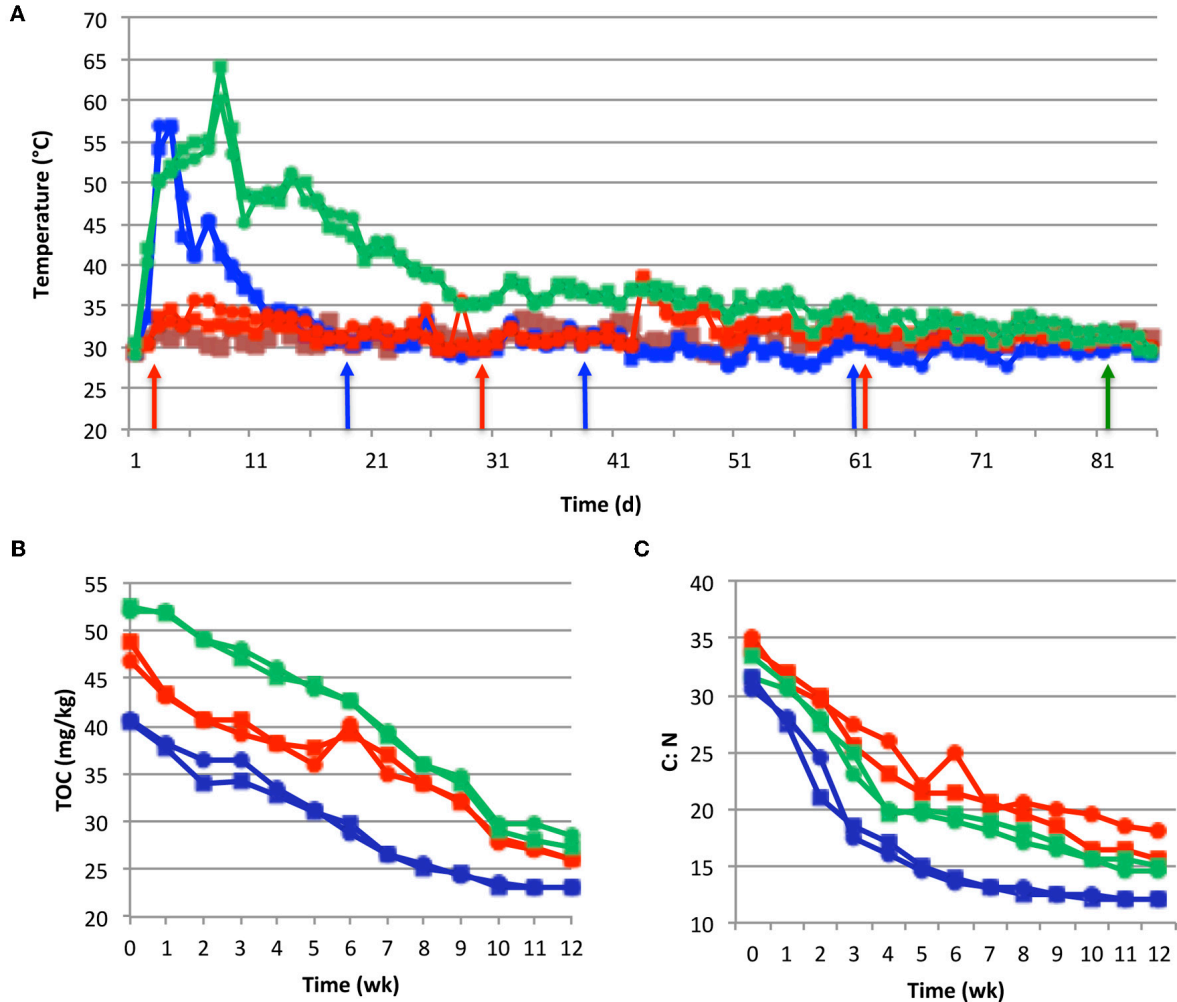
**Compost physico-chemical dynamics. Panel A**, temperature; **Panel B**, total organic carbon (TOC); **Panel C**, C:N. All plots are coded as: blue, rice; red, bagasse; green, coffee; squares, cow manure; circles = sheep manure. In **Panel A**, ambient temperature is plotted as a brown line, and arrows indicate approximate times of peaks in nitrification pulses (see Figure 2D). All points are averages of triplicate values. Samples taken at day 0 and day 82 were used for DNA extraction, clone library construction and qPCR analyses. Additional samples were taken at day 2 (rice) and day 3 (coffee) for DNA extraction and qPCR analyses only.

**Figure 2:**
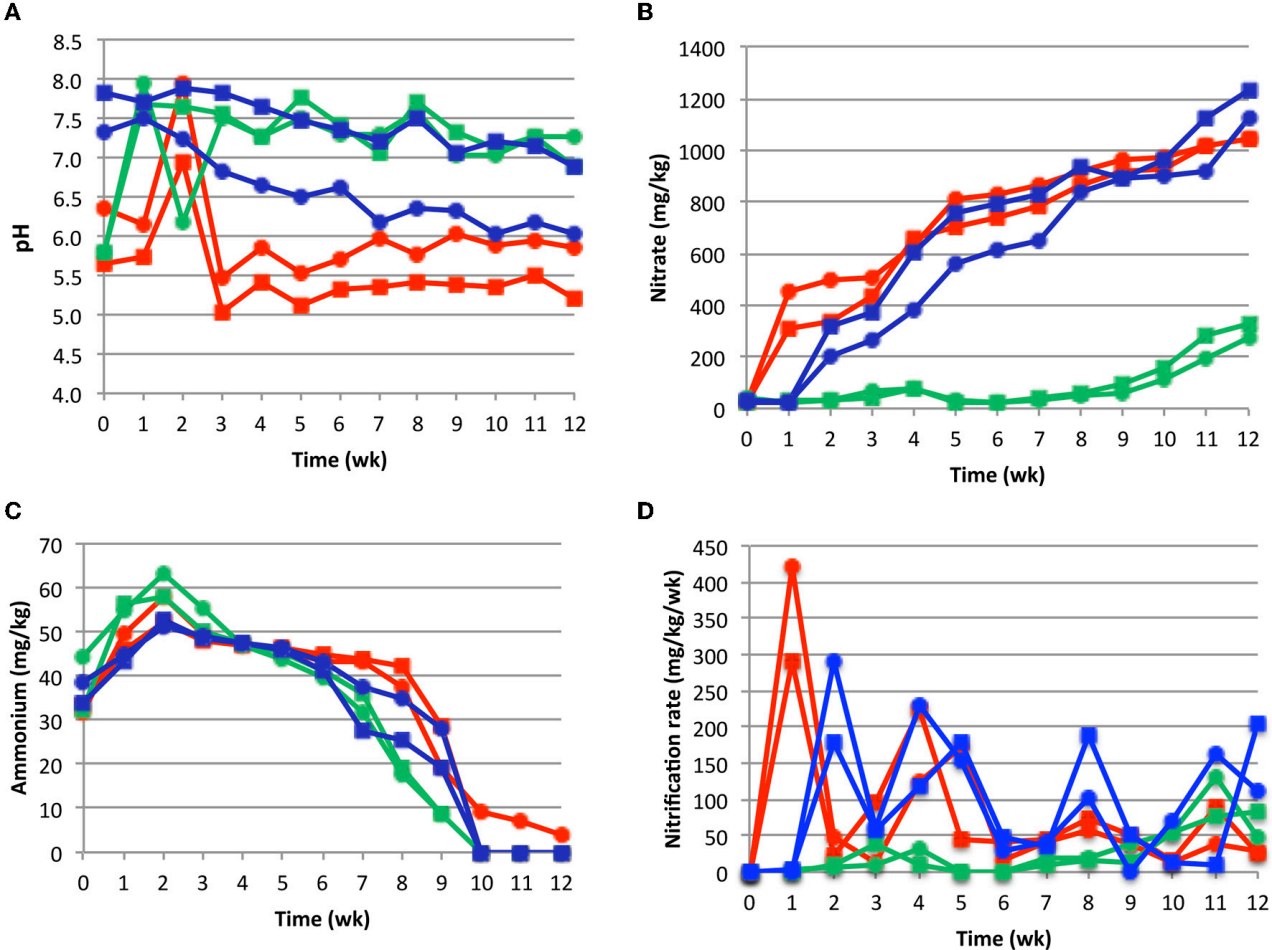
**Compost physiochemical dynamics.** Panel **A**, pH; Panel **B**, nitrate concentration; Panel **C**, ammonium concentration; Panel **D**, apparent nitrification rate. The latter was calculated as the difference in nitrate concentration between two consecutive weekly measures (e.g., [nitrate]_wk2_ − [nitrate]_wk1_). All plots are coded as described for Figure 1.

All composts except for bagasse, reached thermophilic temperatures (≥50°C). In the rice systems, temperatures peaked at *ca*. 57°C (Days 3–4), and then cooled reaching ambient levels (*ca*. 31°C) by day 12 (Figure 1A). Maximum temperature in the coffee (*ca*. 64°C) was attained on day eight, followed by slow cooling to ambient over a period of *ca*. 30 d (Figure 1A). While bagasse lacked a thermophilic phase, microbial activity was evident by periodic temperature spikes of up to 38°C, as well as reductions in TOC (Figure 1B) and C:N (Figure 1C) occurring at rates similar to that of rice and coffee. Each compost had a unique pH profile (Figure 2A). Coffee and bagasse both initially had a pH of 5–6, which spiked to pH of 7–8 within 3 week. These composts then diverged, as pH of the coffee remained in 7–7.5 range for the remainder of the study while, by Week 4, the bagasse had returned to pH 5–6. In contrast, the pH of rice was initially high and dropped steadily.

The composts also differed in the dynamics of N transformations. The bagasse and rice systems had significant nitrate accumulations by Week 2–3, which increased throughout the course of the study (Figure 2B). In contrast, nitrate did not begin to accumulate in coffee until week 11. In all composts, ammonium concentrations (Figure 2C) peaked at Week 3, and at levels that were *ca*. ten-fold lower than those of nitrate. Ammonium then dropped over the course of the study and, by week 11, was below detectable amounts in most of the treatments. Each compost system displayed a unique pattern of apparent nitrification (Figure 2D). Bagasse and rice were similar in that nitrification was pulsed. However, in rice, the maximum nitrification rate of each pulse was similar while, in bagasse, these rates attenuated following an initially high burst of activity. However, the latter occurred within one week, while in rice the initial nitrification pulse was in week 2, and followed compost cooling (Figures 1A and 2D). In coffee, the apparent onset of nitrification was delayed, and also appeared to occur following the extended cooling period (Figures 1A and 2D). All N transformations were significantly affected by plant material, while the (N) source (manure type) only affected ammonium levels (Table A1).

**Figure 3:**
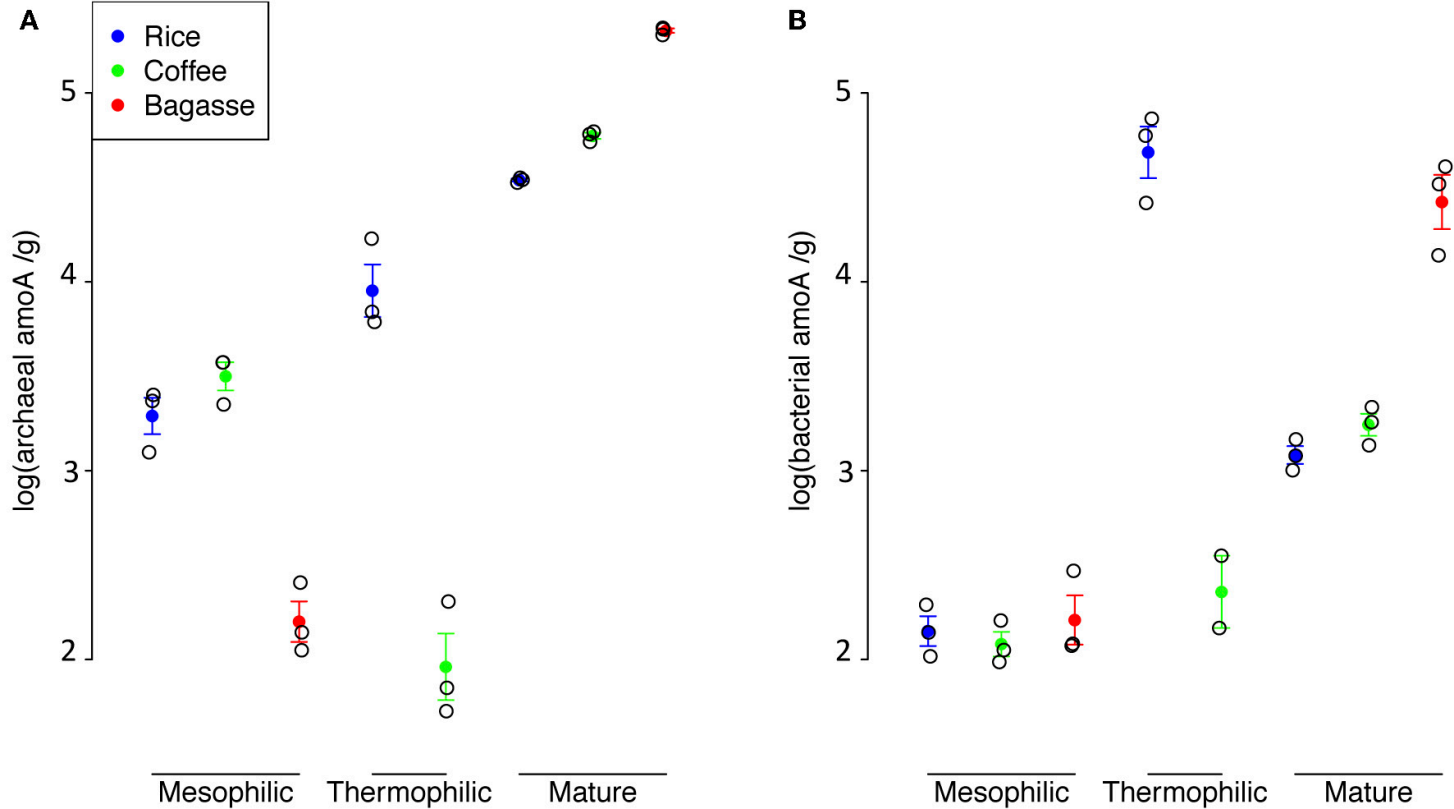
**Abundance of archaeal *amoA* genes (Panel A) and bacterial *amoA* genes (Panel B) in each compost as a function of phase.** Solid circles are the average values for triplicate qPCR runs, open circles are values for an individual qPCR run and bars indicate the standard error.

Abundance and population dynamics of AOA differed significantly as a function of compost type and phase (Figure 3A). In the mesophilic phase, AOA abundance in rice and coffee systems was similar and significantly greater (*p* < 0.00001) than that in bagasse. But, by the end of the study, the AOA population in bagasse had expanded by three orders of magnitude and was significantly greater than that of coffee (*p* < 0.01) or rice (*p* < 0.0006). The thermophilic phase had differing effects on AOA abundance: In the coffee compost, AOA numbers dropped significantly (*p* < 0.000002) while in rice, there was a significant (*p* < 0.003) increase in AOA abundance.

In the majority of cases, the abundance of AOA was significantly greater (*p* < 0.001–0.0000001) than that of AOB (Figures 3A,B). The exceptions were the mesophilic phase of the bagasse and thermophilic phase of the coffee, in both of which AOA *vs*. AOB abundance was not significantly different, and the rice thermophilic phase for which AOB abundance was significantly greater (*p* < 0.001) than that of AOA. The bagasse compost showed the greatest shift in AOA *vs*. AOB abundance: in the mesophilic phase, populations of these groups were numerically similar, but in the mature phase AOA abundance were two orders of magnitude greater than that of AOB (*p* < 0.0000001). In the thermophilic phase of the rice compost, AOB abundance the increased significantly (*p* < 0.0000001) by *ca*. three orders of magnitude relative to the AOB numbers in the mesophilic phase.

A total of 211 archaeal *amoA* sequences were obtained from the ten clone libraries and the best Genbank matches to these were AOA *amoA* sequences from a diversity of environments (Tables A2–A4). The two most common matches were an uncultured thaumarchaeota from a (N) rich wetland (Wang et al., 2011) and a clone from cattle manure compost (Yamamoto et al., 2010). Others were archaeal *amoA* sequences from: temperate soils (Onodera et al., 2010) landfill cover soils (Im et al., 2011), coastal ground water systems (Rogers and Casciotti, 2010), creeks (Herrmann et al., 2011), activated saline sludge and estuaries (Mosier and Francis, 2008; Wankel et al., 2011), Notably, four clones unique to bagasse composts had the highest similarity (98%) to *amoA* from a cultured soil AOA, *Nitrososphaera viennensis* (Tourna et al., 2011).

**Figure 4:**
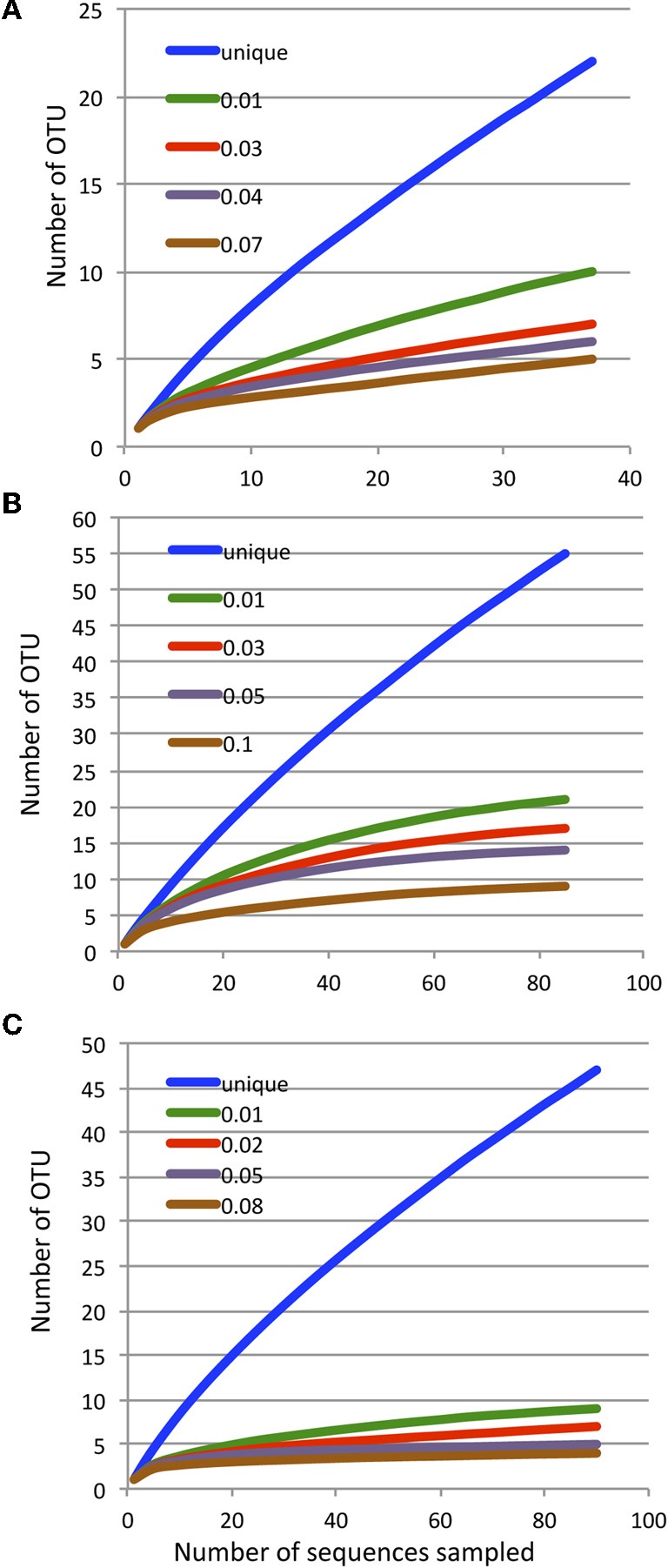
**Rarefaction analyses of the archaeal *amoA* libraries from the rice (Panel A), bagasse (Panel C) and coffee (Panel C) composts.** For the bagasse and rice composts, cow- and sheep-manure treatments were pooled. Each curve illustrates the cumulative OTU number at the indicated phylogenetic distance.

Rarefaction analyses of each library yielded asymtotic curves when phylotypes were defined at any distance ≥0.01 (Figures 4A–C). For any given plant waste type, there were no discernable differences in the composition of clone libraries made from the sheep- or cow-manure treatments (Table A4). Thus, sequences were pooled by plant waste material for these analyses. A distance of 0.02 used to define phylotypes, yield 24 operational taxonomic units (OTUs). Collectively, OTU 1 and OTU 2 comprised *ca*. 68% (143) of the total clone library, and contained representatives of all composts from both the mesophilic and mature stages. Six OTUs were present in both phases, while 10 and 8 OTUs were unique to the mesophilic and mature phases, respectively. Only OTU 1, 2, and 5 were common to all compost types. Half of the OTUs were unique to the bagasse mesophilic and mature stages. The coffee compost had the lowest number of total and unique OTU, despite the fact that it had the single largest clone library (Figure 4B).

**Figure 5:**
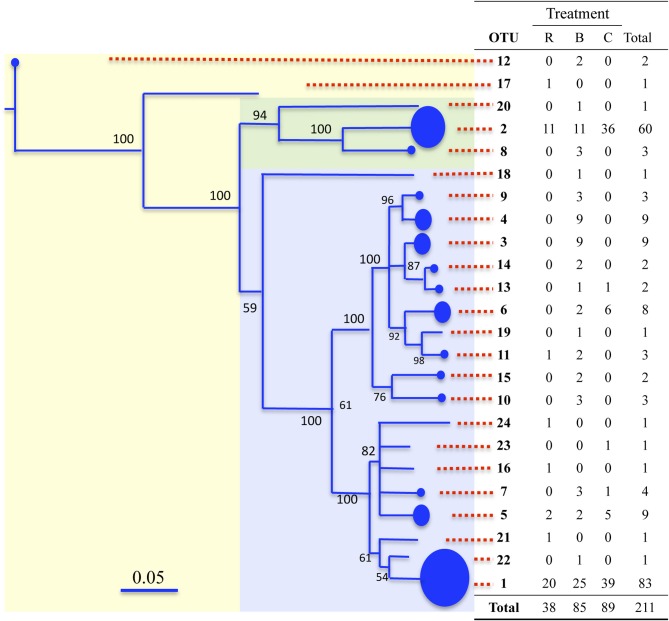
**Phylogram of archaeal *amoA* sequences from the composts.** The two main branches are indicated by the light green and blue areas. Each tip is an OTU, and the OTU number is given in the table. The size of circles at the tips is proportional to the number of sequences comprising that OTU, which are given in the table. Bare tips indicate single sequences. Abbreviations in the table are: R, rice; B, bagasse and C, coffee. In the latter two, the sheep and cow manure treatments are pooled. Bootstrap values (1000 replicates) are given at nodes.

**Figure 6:**
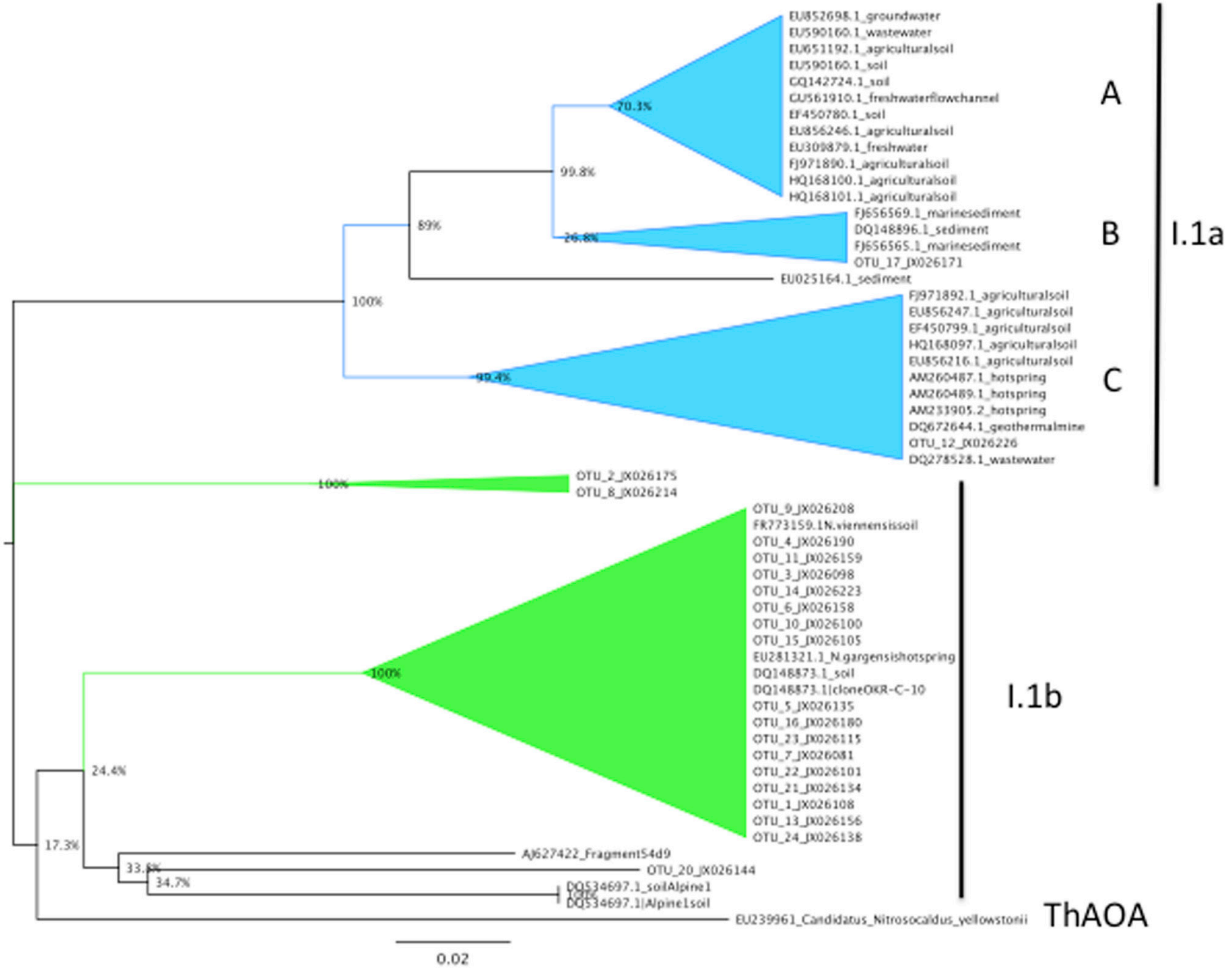
**Neighbor-joining tree of archaeal *amoA* sequences from the composts and reference sequences from the archaeal lineages I.1a (subgroups A, B, and C), I.1b and the thermophilic AOA (ThAOA).** Bootstrap values (1000 replicates) are given at nodes.

A phylogram revealed the archaeal *amoA* sequences to form a tree with three branches (Figure 5). Two branches were formed by single OTUs (OTU 12 and 17, respectively), and the rest of the OTU grouped together on a third branch (Figure 5). There were two sub-groups on the third branch. One large cluster contained OTU 1 along with 18 of the other OTUs. Six of the OTUs in this cluster were composed entirely of sequences from the bagasse compost, and one clade contained sequences only from the mature phase of that system. The other subgroup was formed by OTUs 2, 8, and 20. All phylotypes were of archaeal group I.1b, except OTUs 12 and 17, which were both group I.1a (Figure 6).

**Figure 7:**
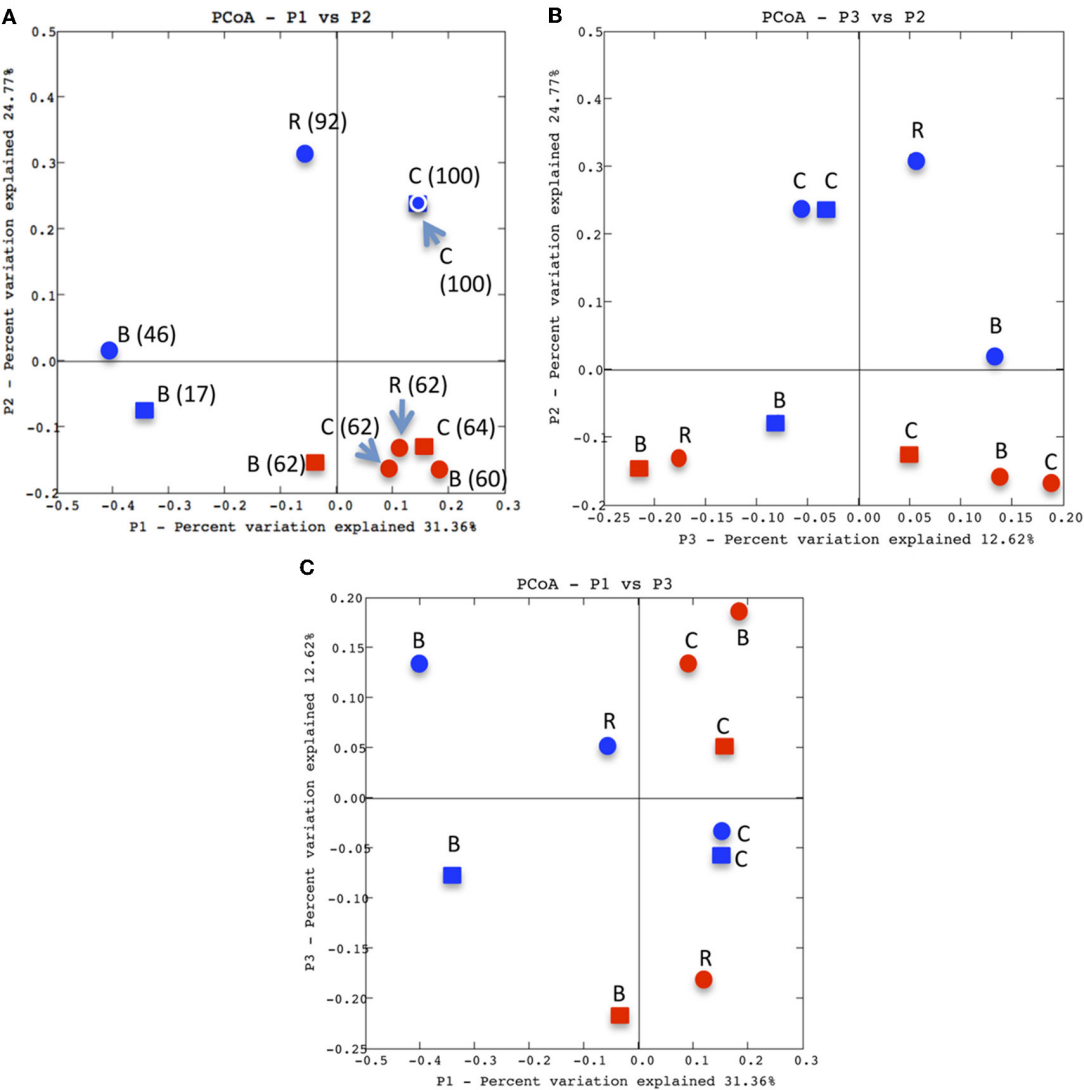
**Unifrac PCA analyses of archaeal *amoA* libraries.** Panels **A–C** are: P1 *vs*. P2, P3 *vs*. P2 and P1 *vs*. P3, respectively. Samples are coded as: mesophilic, red, mature, blue, sheep manure, circles, cow manure, squares, R, rice, B, bagasse and C, coffee. In Panel **A**, values in parentheses are the percentage of sequences from the indicated library that were OTU 1 or OTU 2.

Results of PCA showed P1 and P2 collectively accounted for *ca*. 56% of the variation in the data (Figure 7A). A plot of P1 *vs*. P2 separated the AOA communities primarily by phase, with most mesophilic-phase samples clustered in one quadrant and the mature phase communities distributed across the other three quadrants. A key difference between the mesophilic- and mature-phase communities was the relative abundance of OTUs 1 and 2, which was similar in all mesophilic-phase communities (60–64% of the total sequences in a given sample), but divergent in the mature phase (Figure 7A). In the latter, the archaeal *amoA* libraries of the coffee composts were composed entirely of OTUs 1 and 2, while in bagasse these OTUs, were 17–46% of the total sequences in the respective samples. While the mature phase AOA communities were dispersed across the quadrants in the P1 *vs*. P2 plot, samples of a common plant waste type (*i.e*., bagasse or coffee) remained closely associated. A plot of P2 *vs*. P3 also separated the mesophilic- from mature-phase communities, but there was no strong clustering by phase as in the P1 *vs*. P2 plot. The strongest treatment effect illustrated by the PCA plots was the consistent close association of the mature-phase coffee communities (Figures 7A–C). None of the three PCA contrasts showed any strong effect of manure type used in the composts on the AOA communities.

## Discussion

Population densities of AOA varied by compost type and phase. But, generally, abundance of AOA significantly exceeded that of AOB. Yamamoto et al. (2010) reported varying numbers of AOA *vs*. AOB during composting of cow manure and AOA were predominant by the end of the process. Zeng et al. (2011) examined AOA *vs*. AOB in a compost mixture of rice straw, bran, vegetables and soil; numbers of AOB exceeded AOA for 10 d at the beginning and end of the 50-d process. However, only AOA were detectable during the intervening 30-d period. There are numerous examples from soil and other environments where AOA predominate over AOB (Leininger et al., 2006; Wuchter et al., 2006; Caffrey et al., 2007; He et al., 2007; Nakagawa et al., 2007; Adair and Schwartz, 2008; Beman et al., 2008; Chen et al., 2008; De La Torre et al., 2008; Hatzenpichler et al., 2008; Le Roux et al., 2008; Reigstad et al., 2008; Shen et al., 2008). Yet, the significance of the relative abundance of AOA *vs*. AOB to nitrification remains uncertain (Erguder et al., 2009; Zeglin et al., 2011; Zeng et al., 2011). This is particularly true given the emerging view of soil AOA (group I.1b) as organisms that are potentially metabolically versatile, rather than strict chemolithoautotrophs reliant upon ammonia oxidation for energy generation (Jia and Conrad, 2009; Mertens, 2009; Offre et al., 2009; Bates et al., 2010; Zhang et al., 2010; Blainey et al., 2011; Mussmann, 2011; Xu et al., 2012). Nevertheless, assuming that ammonia oxidation is at least one of the major activities of AOA, their abundance in the composts of the present study, as well as that of prior researchers, could make AOA significant contributors to nitrification in these systems.

Prior to this study, information on the diversity of AOA in composts was restricted to DGGE surveys (Yamamoto et al., 2011; Zeng et al., 2011), which gave valuable initial insights, but very limited sequence data for phylogenetic assessments. The archaeal *amoA* clone library analysis of the present study has thus expanded our depth of knowledge regarding AOA in these environments. In our study, rarefaction analyses of the archaeal *amoA* clone libraries indicated that these provided an adequate sampling of the species richness in the composts. The data illustrated that, while diversity varied as a function of compost type and phase, the AOA communities could be considered relatively diverse. In the context of compost environments, this finding is consistent with that of the DGGE survey of Zeng et al. (2011), but in contrast to that of Yamamoto et al. (2011). Notably, composts comprised substantially of plant materials were examined in the present study and by Zeng and co-workers (2011), while that studied by Yamamoto et al. was cattle manure. Possibly, these patterns in AOA distribution/diversity may indicate that manure, and by extension enteric systems of animals, is a relatively poor habitat for AOA.

Consistent with phylogenetic studies of AOA in other terrestrial systems, nearly all of the AOA phylotypes (22 of 24 total OTUs) belonged to archaeal group I.1b, which is the main soil lineage within the thaumarchaeota (Schleper et al., 2005; Treusch et al., 2005; Jung et al., 2011). While there is an abundance of phylogenetic data on soil AOA, comparing soils and composts to assess similarities or differences in the relative diversity of AOA communities is complicated by variation in soil and compost types, methods for molecular sampling of AOA populations and variation in data reporting (Onodera et al., 2010). Nevertheless, some broad patterns were discernable. For example, in a relatively extensive study of AOA diversity in forest soils, Onodera and co-workers constructed 11 clone libraries and discerned 75 OTUs (at a distance of 0.03) from 448 archaeal *amoA* sequences. A general similarity between the present study and that of Onodera et al. (2010) was that AOA communities were often dominated by one or two phylotypes. Further information on AOA genomics and biology will be needed to interpret such patterns.

As in prior studies with heterotrophic microbes (Peters et al., 2000; Ryckeboer et al., 2003; Charest et al., 2004; Anastasi et al., 2005; Tiquia, 2005; Klamer and Baath, 2006; Partanen et al., 2010), structures of the AOA communities were transformed during the course of the composting process. At the start of the process (mesophilic phase), PCA analysis demonstrated that all AOA communities were broadly similar, and a predominant feature was the prevalence of OTU 1 and 2. But, the community profiles in compost product (mature phase) varied as a function of the plant waste. In the bagasse compost, AOA communities evolved a structure more diverse than it had in the mesophilic phase. In contrast, the mature phase AOA communities of coffee and rice evolved a less diverse structure. The divergence of the relatively low diversity coffee *vs*. high diversity bagasse was particularly evident by the frequent clustering of coffee samples vs. dispersion of bagasse. The AOA in the composts could have, and probably did, originate from both the plant wastes and manures. But, of the two, the plant source appeared to have a more significant effect as PCA analyses indicated by more frequent clustering by plant material type than by manure type. Additionally, AOA abundance was initially significantly greater in coffee compost than it was in bagasse system; if manure were the primary source of AOA, these patterns should have been reversed, as the amount of manure added to the coffee compost was more than 3-fold greater than that used to compose the bagasse system. Furthermore, prior studies of cow- or cattle-manure composts have found AOA to be either non-detectable or their communities to have relatively low diversity (Maeda et al., 2010; Yamamoto et al., 2010). Thus, as noted above, manure appears to be a poor habitat for AOA. The plant wastes used originated from different regions of Trinidad, and were grown on different soil types. Thus, variation in AOA communities from those areas could have been a factor affecting the initial variability in AOA communities of the composts.

The bagasse compost was distinguished by the absence of a thermophilic phase, which could have been a key factor affecting the divergent evolution of its AOA community. In the mesophilic phase, OTU 1 was the predominant phylotype only in the bagasse. But, at the mature stage, this pattern was reversed, and in the bagasse OTU 1 was supplanted by multiple, subordinate phylotypes. Possibly, the latter were thermally sensitive and/or sensitive to other biological and/or physiochemical aspects of the thermophilic stage to which OTU 1 was tolerant. Regardless of the mechanism, these subordinate phylotypes appeared to proliferate greatly in the bagasse, as indicated by the three order of magnitude increase in AOA densities in the mature- *vs*. mesophilic-phases of that compost.

The mature phase coffee composts were distinguished in having comparatively low diversity and this could have been due to having a longer curing process than rice following the thermophilic phase. While it had the largest clone library, AOA diversity was lower than that of the rice system, which had a sequence library less than half the size of the coffee. The coffee composts were also notable in being the major source of OTU 2, the abundance of which was comparable to that of OTU 1 in both phases. Thus, while the relation of phylotype to phenotype are unknown, it is possible the prevalence of OTU 2 in the coffee system might have reflected a physiological characteristic of that group that was better suited to the environment of the coffee composts than to either of the other two.

The type of plant material used in the compost was a key factor in the dynamics of nitrification, and compost behavior in general. A fundamental aspect was the effect on the self-heating process, specifically the divergent behavior of the bagasse. The reason(s) for the lack of a thermophilic phase in the bagasse are unknown, but one possible explanation could be the variation in the lignocellulose content. But, the lignocellulose content of the coffee was similar to that of bagasse, yet coffee exhibited the most pronounced thermophilic phase. Hence another factor might have been the variation between the plant materials in particle size. Notably, bagasse had the largest particle size (lowest surface area) of the materials used in the compost, which could have resulted in slower biodegradation rates, and consequently less heat (Barrington et al., 2002). The larger particle size of bagasse could have also affected porosity in a manner that facilitated release of heat from the pile.

The effects of the compost self-heating process on ammonia-oxidizing microbes and nitrification are not well understood. In the present study, the effect(s) varied between compost types, which differed in terms of the maximal temperature attained, and in the duration of the thermophilic phase (>40°C). In the rice system (maximal temp., *ca*. 57°C, thermophilic phase duration, *ca*. 8 d) abundance of both AOA and AOB was significantly greater than in the mesophilic phase, and the apparent increases in both ammonia-oxidizer communities was followed by the first and largest peak in the apparent nitrification rate in those composts. In contrast, in the thermophilic phase of the coffee composts (maximal temp, *ca*. 65°C, thermophilic phase duration, *ca*. 30 d), the AOA showed a significant decline while AOB showed no significant change in abundance. Nitrification however, was limited until nearly the conclusion of the study, at which time AOA and AOB numbers had increased significantly relative to the mesophilic phase.

Other studies of compost have also yielded conflicting data concerning the effects of temperature on nitrification in these systems. (Sanchez-Monedero et al., 2001) reported an apparent absence of nitrification at elevated temperatures (*ca*. >40°C), while Jarvis et al. (2009) found nitrification occurred throughout the process, including periods of elevated temperature. Divergent results were also obtained from prior studies in which qPCR was applied to monitor nitrifier abundance. The data of Zeng et al. (2011) show AOA were abundant from the start to finish of the compost study, and compost self-heating had little, if any, effect on AOA abundance. But, AOB were quantifiable only prior to compost heating, and again after heating had subsided. In contrast, data from Yamamoto and co-workers (2010, 2011) showed apparent proliferation of both AOB and AOA at elevated temperatures. Lastly, Maeda et al. (2010) were unable to detect AOA at any time in their study of a cow manure compost. But, high numbers of AOB and nitrification were consistently detected on the surface of the pile, where nitrification was active throughout the study and at temperatures approaching 70°C. A meaningful interpretation of these diverse patterns will require a better understanding of the physiology and ecophysiology of both AOB and AOA.

## Conclusions

The present study demonstrated that AOA were relatively abundant in composts of tropical agricultural wastes, and significantly more so than were the AOB. Furthermore, while the AOA communities in the composts were predominantly group I.1b, the communities were diverse and exhibited structures that diverged between compost types and phases. Variations in AOA community structure developed in parallel with physico-chemical evolution processes that were unique to each compost. These coincident patterns could be taken as indicators of the ecophysiological diversity in the soil AOA (group I.1b), in that significantly different AOA communities developed when exposed to varying physico-chemical environments. Nitrification patterns and levels differed in the composts which, for the mature material, could have significant effects on its performance as a plant growth medium. Thus, it will also be important to determine the association of AOA (and diversity in their communities) with nitrification in these systems.

### Conflict of interest statement

The authors declare that the research was conducted in the absence of any commercial or financial relationships that could be construed as a potential conflict of interest.

## Appendix

**Table A1:** **Statistical analyses for carbon and nitrogen substrates and interactive effects of bothsubstrates on physico-chemical parameters throughout the composting process**.

**Parameters analyzed**	**Substrate Type**	***P* values**	
		**Main effects**	**Interactive effect**
pH	Carbon	<0.001	
	Nitrogen	<0.001	
	Carbon^*^Nitrogen		0.001
TOC	Carbon	<0.001	
	Nitrogen	0.726	
	Carbon^*^Nitrogen		0.523
C:N	Carbon	<0.001	
	Nitrogen	0.444	
	Carbon^*^Nitrogen		0.108
AMM	Carbon	<0.001	
	Nitrogen	<0.001	
	Carbon^*^Nitrogen		0.029
NIT	Carbon	<0.001	
	Nitrogen	0.137	
	Carbon^*^Nitrogen		0.007
TN	Carbon	<0.001	
	Nitrogen	0.8	
	Carbon^*^Nitrogen		0.112
Temperature	Carbon	<0.001	
	Nitrogen	<0.001	
	Carbon^*^Nitrogen		<0.001

**Table A2:** **Distribution of OTU by compost phase and treatment**.

**OTU**	**Mesophilic phase**				**Mature phase**				**Grand total**
	**Rice**	**Bagasse**	**Coffee**	**Total**	**Rice**	**Bagasse**	**Coffee**	**Total**	
1	4	21	10	35	16	4	29	49	83
2	4	6	14	24	7	7	22	36	60
3	0	4	0	4	0	5	0	5	9
4	0	0	0	0	0	9	0	9	9
5	2	1	5	8	0	1	0	1	9
6	0	2	6	8	0	0	0	0	8
7	0	3	1	4	0	0	0	0	4
8	0	3	0	3	0	0	0	0	3
9	0	0	0	0	0	3	0	3	3
10	0	1	0	1	0	2	0	2	3
11	1	2	0	3	0	0	0	0	3
12	0	0	0	0	0	2	0	2	2
13	0	0	1	1	0	1	0	1	2
14	0	0	0	0	0	2	0	2	2
15	0	2	0	2	0	0	0	0	2
16	0	0	0	0	1	0	0	1	1
17	0	0	0	0	1	0	0	1	1
18	0	0	0	0	0	1	0	1	1
19	0	1	0	1	0	0	0	0	1
20	0	1	0	1	0	0	0	0	1
21	1	0	0	1	0	0	0	0	1
22	0	1	0	1	0	0	0	0	1
23	0	0	1	1	0	0	0	0	1
24	1	0	0	1	0	0	0	0	1
Total	13	48	38	99	25	37	51	113	211

**Table A3:** **Genbank accession numbers and similarity to mesophilic sequences and OTU**.

**Genbank accession #**	**Genbank similarity**	**References**	**OTU**
HQ538551.1	97%	Wang et al., 2011	1, 2, 3, 4, 6
	98%		
AB542171.1	100%	Yamamoto et al., 2010	2, 23
	99%		
HM16492.1	96%	Im et al., 2011	21
HQ678192	97%	Xia et al., 2011	21
AB542178.1	99%	Yamamoto et al., 2010	7
	100%		
AB542175.1	100%	Yamamoto et al., 2010	5, 24
	99%		
HM589849.1	96%	Wu et al., (unpublished)	11, 19
	98%		
HM164866.1	100%	Im et al., 2011	15
HM160464	93%	Rogers and Casciotti, 2010	6, 22
JF799755.1	100%	Xu et al., 2010 unpublished	3, 13
	94%		
HM363828.1	99%	Wankel et al., 2011	20

**Table A4:** **Genbank accession numbers and similarity to mature sequences and OTU**.

**Genbank accession #**	**Genbank similarity**	**References**	**OTU**
HQ538551	97%	Wang et al., 2011	1
	98%		
	99%		
	100%		
AB542171	98%	Yamamoto et al., 2010	2, 16
	99%		
	100%		
EU651256.1	98%	Mosier and Francis (2008)	17
HQ678192	98%	Xia et al. (2011)	1
FR773159	98%	Tourna et al. (2011)	4, 9
	99%		
JF500154	97%	Gong et al, unpublished	10
JF799754	96%	Xu et al., 2010 unpublished	9, 14
	98%		
HQ677701	100%	Wu et al, unpublished	3, 8
HQ678192	99%	Xia et al. (2011)	
HQ401426	99%	Herrmann et al., 2011	18
AB529739	89%	Onodera et al. (2010)	12
HM164921	98%	Im et al. (2011)	1
